# Supplementary material for: Email Reminders Increase the Frequency That Pet Owners Update Their Microchip Information
Source: Animals (Basel). 2018 Jan 31;8(2):20. doi: 10.3390/ani8020020 (PMC5836028; doi:10.3390/ani8020020)
Supplement: Supplementary file 1 [file animals-08-00020-s001.pdf]

animals

MDPI

Article

# Email Reminders Increase the Frequency That Pet Owners Update Their Microchip Information

Katie Goodwin [1,*], Jacquie Rand [1,2], John Morton [1,3], Varun Uthappa [4] and Rick Walduck [4]

Supplementary Materials

Table S1. Microchipping regulations in Australian states and territories [1-10].

State/Territory	Microchip Requirements
Australian Capital Territory	Microchipping of cats and dogs by 12 weeks and prior to sale or transfer of ownership.<br>Details recorded:<br>• Pet: breed, date of birth, color, sex, neuter status, address, dangerous status<br>• Owner: name, address, contact phone number, alternate contact name and phone number<br>• Implanter: name and business address
New South Wales	Microchipping of cats and dogs by 12 weeks and prior to sale or transfer.<br>Details recorded:<br>• Pet: species, breed, date of birth, sex, color, markings, address, municipality<br>• Owner: name, address and contact phone number
Northern Territory	Not compulsory, except in one local government (council) area
Queensland	Microchipping of cats and dogs by 12 weeks and prior to sale or transfer
South Australia	Not compulsory until 2018, except in some local government (council) areas
Tasmania	Microchipping of cats and dogs by 6 months
Victoria	Microchipping of cats and dogs by 3 months, at the time of compulsory council registration. Microchipping prior to sale or transfer for domestic animal businesses.<br>Details recorded:<br>• Pet: species and breed, date of birth, color, sex, neutered status, address, municipality, dangerous status<br>• Owner: name, address and phone number, alternate contact name and phone number.<br>• Implanter: name and business address
Western Australia	All dogs must be microchipped. Cats must be microchipped by 6 months and prior to sale or transfer.

Table S2. Sex of new cats registered on the CAR microchip database, by year of implantation. Cats with no sex recorded make up the remaining %.

Year Implanted	Male	%	Female	%	Male	%	Female	%	Male	%	Female	%	Male	%	Female	%
State	ACT				NSW				NT				QLD			
2008	250	53.30	215	45.84	550	48.54	565	49.87	214	47.56	233	51.78	2559	48.93	2629	50.27
2009	193	46.62	219	52.90	531	48.49	551	50.32	196	48.40	202	49.88	3629	47.84	3899	51.40
2010	197	51.98	175	46.17	449	47.56	480	50.85	124	50.82	117	47.95	3196	47.93	3387	50.79
2011	225	46.30	255	52.47	416	49.94	410	49.22	109	49.10	111	50.00	2940	49.18	2998	50.15
2012	166	49.55	166	49.55	372	47.39	409	52.10	123	49.00	128	51.00	2542	48.48	2663	50.79
2013	161	51.60	148	47.44	409	51.32	384	48.18	135	49.45	136	49.82	1648	48.14	1751	51.15
2014	178	45.64	211	54.10	421	49.36	428	50.18	123	46.07	143	53.56	1684	49.46	1706	50.10
2015	261	50.10	257	49.33	364	49.39	371	50.34	290	50.79	275	48.16	1810	49.66	1818	49.88
2016	216	50.94	206	48.58	441	49.16	452	50.39	273	45.65	318	53.18	1988	49.66	2002	50.01
Average	205.22	49.56	205.78	49.60	439.22	49.02	450	50.16	176.33	48.54	184.78	50.59	2444	48.81	2539.22	50.51
State	SA				Tas				Vic				WA			
2008	1525	50.08	1491	48.97	282	50.63	271	48.65	15,887	48.50	16,564	50.57	1301	52.10	1170	46.86
2009	1728	50.50	1673	48.89	623	46.32	709	52.71	15,585	49.16	15,715	49.57	1483	51.49	1379	47.88
2010	1556	47.47	1690	51.56	705	50.39	678	48.46	14,063	49.41	14,017	49.24	1210	50.84	1140	47.90
2011	1567	49.09	1602	50.19	729	49.49	729	49.49	13,058	49.88	12,930	49.39	1151	50.42	1119	49.01
2012	1369	49.67	1377	49.96	616	50.99	580	48.01	13,892	48.78	14,393	50.54	1192	49.92	1182	49.50
2013	1180	48.82	1228	50.81	573	50.44	555	48.86	15,635	49.33	15,878	50.09	2600	47.96	2778	51.25
2014	820	49.82	818	49.70	412	52.42	370	47.07	15,962	49.04	16,419	50.44	1214	48.76	1254	50.36
2015	680	50.60	655	48.74	318	48.40	330	50.23	15,950	49.24	16,255	50.18	1249	47.65	1363	52.00
2016	1410	46.02	1643	53.62	377	53.93	317	45.35	15,398	49.23	15,679	50.13	1301	49.90	1285	49.29
Average	1315	49.12	1353	50.27	515	50.34	504.33	48.76	15,047.78	49.18	15,316.67	50.02	1411.22	49.89	1407.78	49.34

Table S3. Sex of new dogs registered on the CAR microchip database, by year of implantation. Dogs with no sex recorded make up the remaining %.

Year Implanted	Male	%	Female	%	Male	%	Female	%	Male	%	Female	%	Male	%	Female	%
State	ACT				NSW				NT				QLD			
2008	590	52.12	531	46.91	1809	49.29	1819	49.56	983	49.10	1004	50.15	6681	50.80	6358	48.35
2009	577	51.38	542	48.26	1567	49.06	1593	49.87	825	49.82	813	49.09	9574	49.72	9518	49.43
2010	478	52.93	410	45.40	1417	48.98	1429	49.40	724	50.14	692	47.92	8859	49.92	8561	48.24
2011	510	51.88	465	47.30	1252	48.72	1288	50.12	878	51.59	816	47.94	6276	50.15	6166	49.27
2012	409	53.96	343	45.25	1320	48.64	1378	50.77	621	49.01	638	50.36	5942	50.44	5733	48.66
2013	367	50.62	352	48.55	1326	49.00	1369	50.59	597	47.38	657	52.14	4404	50.21	4322	49.28
2014	452	52.50	405	47.04	1320	49.44	1342	50.26	679	51.99	622	47.63	4409	50.56	4247	48.70
2015	443	52.74	390	46.43	1194	48.18	1268	51.17	792	49.72	791	49.65	4625	50.06	4541	49.16
2016	421	50.48	408	48.92	1265	48.17	1349	51.37	881	51.43	823	48.04	5802	49.90	5779	49.70
Average	471.89	52.07	427.33	47.12	1385.56	48.83	1426.11	50.35	775.56	50.02	761.78	49.21	6285.78	50.20	6136.11	48.98
State	SA				Tas				Vic				WA			
2008	4519	51.06	4280	48.36	1630	48.28	1713	50.74	39,320	50.53	37,899	48.70	4620	50.91	4387	48.34
2009	4378	50.49	4218	48.64	1685	48.56	1760	50.72	38,449	50.67	36,712	48.38	4391	51.84	4007	47.31
2010	4365	50.78	4097	47.66	4393	49.04	4472	49.92	34,302	50.38	32,808	48.19	4235	52.21	3785	46.66
2011	3169	49.94	3146	49.57	8622	50.08	8472	49.21	30,605	50.95	29,106	48.45	3951	51.88	3637	47.76
2012	3328	50.72	3187	48.57	2947	50.57	2836	48.67	31,688	50.96	30,130	48.46	3266	51.38	3028	47.63
2013	3082	51.05	2931	48.55	2555	51.03	2412	48.17	29,053	50.67	28,010	48.85	3417	51.71	3159	47.81
2014	2234	49.92	2215	49.50	2081	51.32	1943	47.92	27,265	51.48	25,507	48.16	3600	51.37	3361	47.96
2015	2307	51.11	2187	48.45	1722	50.72	1640	48.31	25,654	51.06	24,350	48.46	5931	49.99	5836	49.19
2016	2987	52.25	2714	47.47	1955	50.87	1860	48.40	25,522	51.26	24,063	48.33	5074	50.30	4935	48.92
Average	3374.33	50.81	3219.44	48.53	3065.56	50.05	3012	49.12	31,317.56	50.88	29,842.78	48.44	4276.11	51.29	4015	47.95

Table S4. Numbers of new cat registrations on the CAR microchip database by year of implantation, according to state or territory of residence listed as at 17 August 2017.

Year Implanted	No. Cats	Proportion of Cats in State/ Territory (%)	Cats/1000 Residents	Human Population	No. Cats	Proportion of Cats in State/ Territory (%)	Cats/1000 Residents	Human Population	No. Cats	Proportion of Cats in State/ Territory (%)	Cats/1000 Residents	Human Population
State	ACT				NSW				NT			
2008	469	1.02	1.35	347,800	1133	2.46	0.16	7,041,400	450	0.98	2.02977	221,700
2009	414	0.85	1.17	354,900	1095	2.24	0.15	7,191,500	405	0.83	1.778656	227,700
2010	379	0.87	1.05	361,900	944	2.16	0.13	7,272,200	244	0.56	1.061331	229,900
2011	486	1.20	1.31	370,700	833	2.05	0.11	7,247,700	222	0.55	0.95525	232,400
2012	335	0.81	0.88	379,600	785	1.89	0.11	7,348,900	251	0.61	1.059519	236,900
2013	312	0.69	0.81	384,100	797	1.75	0.11	7,465,500	273	0.60	1.125309	242,600
2014	390	0.92	1.01	387,600	853	2.01	0.11	7,565,500	267	0.63	1.092919	244,300
2015	521	1.23	1.33	393,000	737	1.73	0.10	7,670,700	571	1.34	2.340164	244,000
2016	424	0.97	1.04	406,400	897	2.06	0.12	7,797,800	598	1.37	2.440816	245,000
Average	414.44	0.95	1.10	376,222.22	897.11	2.04	0.12	7,400,133.33	364.56	0.83	1.54	236,055.56
State	QLD				SA				Tas			
2008	5230	11.34	1.20	4,349,500	3045	6.60	1.89	1,612,000	557	1.21	1.11	500,300
2009	7585	15.53	1.70	4,473,000	3422	7.01	2.09	1,633,900	1345	2.75	2.66	505,400
2010	6668	15.24	1.47	4,548,700	3278	7.49	1.99	1,650,400	1399	3.20	2.75	509,300
2011	5978	14.71	1.32	4,513,000	3192	7.85	1.94	1,645,000	1473	3.62	2.88	511,700
2012	5243	12.65	1.14	4,610,900	2756	6.65	1.66	1,662,200	1208	2.91	2.36	512,400
2013	3423	7.53	0.73	4,690,900	2417	5.32	1.44	1,677,300	1136	2.50	2.21	514,000
2014	3405	8.03	0.72	4,750,500	1646	3.88	0.97	1,691,500	786	1.85	1.53	515,200
2015	3645	8.58	0.76	4,808,800	1344	3.16	0.79	1,702,800	657	1.55	1.27	517,400
2016	4003	9.19	0.82	4,883,700	3064	7.03	1.78	1,717,000	699	1.60	1.35	519,100
Average	5020	11.42	1.09	4,625,444.44	2684.89	6.11	1.62	1,665,788.89	1028.89	2.36	2.01	511,644.44
State	Vic				WA				Total Cats		Total %	
2008	32,754	71.00	6.11	5,364,800	2497	5.41	1.13	2,204,000	46,135		100	

2009	31,700	64.90	5.77	5,496,400	2880	5.90	1.27	2,270,300	48,846	100
2010	28,464	65.05	5.10	5,585,600	2380	5.44	1.03	2,317,100	43,756	100
2011	26,180	64.41	4.70	5,574,500	2283	5.62	0.96	2,387,200	40,647	100
2012	28,476	68.71	5.01	5,679,600	2388	5.76	0.97	2,472,700	41,442	100
2013	31,696	69.70	5.47	5,791,000	5421	11.92	2.13	2,550,900	45,475	100
2014	32,550	76.79	5.53	5,886,400	2490	5.87	0.96	2,581,300	42,387	100
2015	32,392	76.24	5.40	5,996,400	2621	6.17	1.01	2,603,900	42,488	100
2016	31,275	71.79	5.01	6,244,200	2607	5.98	1.02	2,567,800	43,567	100
Average	30,609.67	69.84	5.34	5,735,433.33	2840.78	6.45	1.16	2,439,466.67		

* Percentage of newly registered dogs in state/territory, by year of implantation.

Table S5. Numbers of new dog registrations on the CAR microchip database by year of implantation, according to state or territory of residence listed as at 17 August 2017.

Year Implanted	No. Dogs	Proportion of Dogs in State/ Territory (%) *	Dogs/1000 Residents	Human Population	No. Dogs	Proportion of Dogs in State/ Territory (%) *	Dogs/1000 Residents	Human Population	No. Dogs	Proportion of Dogs in State/ Territory (%) *	Dogs/1000 Residents	Human Population
State	ACT				NSW				NT			
2008	1132	0.95	3.25	347,800	3670	3.08	0.52	7,041,400	2002	1.68	9.03	221,700
2009	1123	0.92	3.16	354,900	3194	2.62	0.44	7,191,500	1656	1.36	7.27	227,700
2010	903	0.77	2.50	361,900	2893	2.48	0.40	7,272,200	1444	1.24	6.28	229,900
2011	983	0.90	2.65	370,700	2570	2.36	0.35	7,247,700	1702	1.56	7.32	232,400
2012	758	0.78	2.00	379,600	2714	2.79	0.37	7,348,900	1267	1.30	5.35	236,900
2013	725	0.82	1.89	384,100	2706	3.06	0.36	7,465,500	1260	1.42	5.19	242,600
2014	861	1.05	2.22	387,600	2670	3.25	0.35	7,565,500	1306	1.59	5.35	244,300
2015	840	1.00	2.14	393,000	2478	2.94	0.32	7,670,700	1593	1.89	6.53	244,000
2016	834	0.97	2.05	406,400	2626	3.05	0.34	7,797,800	1713	1.99	6.99	245,000
Average	906.56	0.91	2.43	376,222.22	2835.67	2.85	0.38	7,400,133.33	1549.22	1.56	6.59	236,055.56
State	QLD				SA				Tas			
2008	13,151	11.04	3.02	4,349,500	8851	7.43	5.49	1,612,000	3376	2.84	6.75	500,300

2009	19,256	15.82	4.30	4,473,000	8671	7.12	5.31	1,633,900	3470	2.85	6.87	505,400
2010	17,745	15.20	3.90	4,548,700	8596	7.36	5.21	1,650,400	8958	7.67	17.59	509,300
2011	12,515	11.48	2.77	4,513,000	6346	5.82	3.86	1,645,000	17,216	15.79	33.64	511,700
2012	11,781	12.09	2.56	4,610,900	6561	6.73	3.95	1,662,200	5827	5.98	11.37	512,400
2013	8771	9.92	1.87	4,690,900	6037	6.82	3.60	1,677,300	5007	5.66	9.74	514,000
2014	8721	10.63	1.84	4,750,500	4475	5.45	2.65	1,691,500	4055	4.94	7.87	515,200
2015	9238	10.98	1.92	4,808,800	4514	5.36	2.65	1,702,800	3395	4.03	6.56	517,400
2016	11,627	13.48	2.38	4,883,700	5717	6.63	3.33	1,717,000	3843	4.46	7.40	519,100
Average	12,533.89	12.29	2.73	4,625,444.44	6640.89	6.53	4.00	1,665,788.89	6127.44	6.02	11.98	511,644.44
State	Vic				WA				Total Dogs		Total %	
2008	77,817	65.35	14.51	5,364,800	9075	7.62	4.12	2,204,000	119,074		100	
2009	75,876	62.34	13.80	5,496,400	8470	6.96	3.73	2,270,300	121,716		100	
2010	68,081	58.32	12.19	5,585,600	8112	6.95	3.50	2,317,100	116,732		100	
2011	60,071	55.10	10.78	5,574,500	7615	6.99	3.19	2,387,200	109,018		100	
2012	62,177	63.81	10.95	5,679,600	6357	6.52	2.57	2,472,700	97,442		100	
2013	57,341	64.83	9.90	5,791,000	6608	7.47	2.59	2,550,900	88,455		100	
2014	52,963	64.54	9.00	5,886,400	7008	8.54	2.71	2,581,300	82,059		100	
2015	50,245	59.70	8.38	5,996,400	11,865	14.10	4.56	2,603,900	84,168		100	
2016	49,791	57.74	7.97	6,244,200	10,087	11.70	3.93	2,567,800	86,238		100	
Average	61,595.78	61.30	10.83	5,735,433.33	8355.22	8.54	3.43	2,439,466.67				

* Percentage of newly registered dogs in state/territory, by year of implantation.

References

1. Australian Capital Territory Government, *Domestic animals regulation 2001*. 2017.
2. Government of South Australia, *South Australia Dog and Cat Management Regulations 2017*. 2017.
3. Government of Western Australia, *Laws for responsible cat owners*, Department of Local Government Sport and Cultural Industries, Editor. 2017: Perth.
4. Government of Western Australia, *Laws for dog owners*, Department of Local Government Sport and Cultural Industries, Editor. 2017: Perth. Accessed 12 October 2017.
5. New South Wales Government, *Companion animals regulation 2008*. 2008.
6. NSW Office of Local Government. *Microchipping & Registration*. 2014 12 October 2017]; Available from: https://www.olg.nsw.gov.au/public/dogs-and-cats/information-for-the-community/microchipping-registration.
7. Queensland Government. *Laws for pet owners in Queensland*. 2016 28 June 2016; Available from: https://www.qld.gov.au/families/government/pets/pages/pet-laws.html.
8. RSPCA Australia. *Microchipping*. Available from: https://www.rspca.org.au/campaigns/responsible-pet-owner/micro-chipping.
9. Tasmanian Government Department of Premier and Cabinet, *Dog control act 2000*. 2010: Hobart.
10. Victoria State Government, *Domestic animals regulations 2005*. 2009.

© 2018 by the authors. Submitted for possible open access publication under the terms and conditions of the Creative Commons Attribution (CC BY) license (http://creativecommons.org/licenses/by/4.0/).
